# Performance Characterisation of a New Plaster Composite Lightened with End-of-Life Tyres’ Recycled Materials for False Ceiling Plates

**DOI:** 10.3390/ma15165660

**Published:** 2022-08-17

**Authors:** Manuel Álvarez, Paulo Santos, Paulo Lopes, David Abrantes, Daniel Ferrández

**Affiliations:** 1 Departamento de Tecnología de la Edificación, Escuela Técnica Superior de Edificación, Universidad Politécnica de Madrid, 28040 Madrid, Spain; 2 ISISE, Civil Engineering Department, Rua Luis Reis Santos, Pólo II da Universidade de Coimbra, 3030-788 Coimbra, Portugal

**Keywords:** false ceilings, new plaster-based composites, end-of-life tyres, recycled materials, performance characterisation

## Abstract

Plaster is one of the most used and studied materials in the building process. This paper shows the result of the characterisation of a new plaster-based material enlightened and reinforced with polymers and end-of-life tyres’ recycled materials. As far as end-of-life tyres are a common waste item, this paper offers new recycling possibilities, as well as significant improvements in new building materials. Mechanical, thermal conductivity, sound absorption, fire reaction and environmental impact are studied and analysed. Three different end-of-life tyres’ recycled materials are used, two size rubber and textile fibres. A significant density reduction up to 17% was achieved mainly due to end-of-life materials lower density. Two thermal conductivity measurement methods, heat flux meter and guarded hot plate, were conducted and then compared. A 20% improvement with respect to the reference was achieved in those samples with textile fibre. The two methods’ measurements got a 1% difference in all samples analysed except textile fibre. Thus, this allowed to validate these methods and assure these measurements. Sound absorption was also measured. These materials reached α = 0.32 in high frequencies. Performance in low frequencies were lower. Fire tests led to no ignition results and no fire propagation. Finally, a basic global warming potential impact study based on environmental product declaration (EPD) is conducted. The most relevant result of this study is the potential 20–34% reduction of CO_2_ emissions with the elaboration of these composites.

## 1. Introduction

Of all the materials that are used in the building process, plaster is still today the one that is most demanded [1,2,3]. The success of this material in construction lies in the fact that it is easy to obtain in nature. In addition, the treatment processes to obtain the final material needs less energy and economics resources consumption [4]. In addition, plaster is a circular material since it can be fully reused during almost forever, as it can be transformed again in binder powder [5,6]. This is a characteristic that should be noted, as it contributes directly to the achievement of Sustainable Development Goals 11 and 12, [7,8]. These goals are related with a production and responsible consumption and sustainable cities and communities. Plaster is widely studied and applied to buildings due to several reasons. On the one hand, its versatility to create different shapes and its elegant aesthetic finish let it be applied to many places and make plaster the ideal material for the interior cladding of buildings, both walls and false ceilings. On the other hand, its good mechanical performance and its weight make it the most suitable material for these uses [9].

However, in addition to the mechanical behaviour, its use in buildings requires other characteristics, such as reduced thermal conductivity and adequate acoustic behaviour. The aim of these requirements is to achieve comfort and reduce the energy consumption of buildings, as the building sector is one of the big energy resources consumers in the world [10]. To improve these properties, there are two diverse types of studies in the literature. On the one hand, there are studies that make additions to modify the matrix. Most of the studies try to make the material more porous, provoking reactions in the matrix to create air pores in its interior. In all these studies, a significant reduction in thermal conductivity is achieved [11,12]. However, this leads to a considerable and undesirable reduction in mechanical strength [10,13]. On the other hand, it can be found studios that use wastes and fibres from diverse origin. In this way, there are several studies made with the addition of both, natural and synthetic fibres, trying to improve both mechanical and thermal resistance of the new materials developed. In these studies, the mechanical resistance increases in a significantly way, but thermal and other resistances achieve results similar to the reference values studied [14,15,16,17].

Acoustic performance significatively improves in those studies when the plaster becomes more porous. An acoustic absorption increases of 20% is achieved in these porous materials, especially in high frequencies [18]. Moreover, other studies were carried out using lignin as additives, to check the acoustic absorption [19]. The difference between the values achieved in the different samples tested were not so relevant (α = 0.15–0.30 in high frequencies). This sound absorption depends on the mixture humidity and the additives quantity [20,21].

Regarding waste additions, rubber from end-of-life tyres is one of the most abundant materials produced, with 355 million tyres produced each year [22]. The main problem with this material is its storage. They end up occupying large areas of land, and, due to their flammable characteristics, this is huge problem that is difficult to manage [23].

Despite this, it is a material that is widely used in construction. Most of the studies are focused on mortars and concrete. Due to this lower density, the main property that rubbers give to composites is a weight attenuation. This reduction is significatively higher than conventional aggregates like construction and demolition waste (CDW) or ceramic [24]. The improvement of mechanical characteristics using end-of life tyres rubber has been demonstrated in several studies. Thomas et al. showed that the use of tyre rubber in mortars and concrete provides a better performance against acids attacks, which means that useful life of this material could increase in some environments [25]. Roychand et al. concluded that particle size is a determining factor in both flexural and compressive strength. In this study, a previous solvent-water washing treatment of the rubber resulted in a compressive strength increase. However, the larger the size of the particle, the higher decrease in the bending strength of the plaster [26].

Thus, the use of rubber as an additive is not very well studied in plaster composites. Regarding mechanical performance, Serna et al. showed a significant reduction in the compressive and flexural strength, up to 18%, with fractions from 1–5% volume [27]. Herrero et al. also studied thermal performance, where small particle size additives of 30–40% by weight presented the best results [28].

The aim of this study is to characterize a new plaster-based material with three different recycled materials obtained from end-of-life tyres; two of them are rubber shaped and one textile fibres, to make false ceiling plates with thermal conductivity and acoustic absorption improved. Thus, have a double impact in building sector. On the one hand, it contributes to a resource consumption reduction and energy saving. On the other hand, the use of end-of-life tyres materials as additives in building materials increases useful life span and contributes to the environment protection by reducing this waste.

## 2. Materials and Methods

### 2.1. Materials

The new composite plasters developed in this study uses four different additives classified in two parts: matrix and additives.

#### 2.1.1. Matrix

The following materials were used as matrix elements:

- Plaster: According to UNE-EN-13279-1 [29], controlled gypsum E-35 provided by Placo Saint Gobain [30] is used as main material;
- Water: Canal de Isabel II water was used in this study. There is not any agent that modifies the samples properties [31]. Due to the sodium polyacrylate additives, material workability decreases when the water decreases. For this, an optimal 0.7 water/plaster ratio was set.

#### 2.1.2. Additives

**Figure 1:**
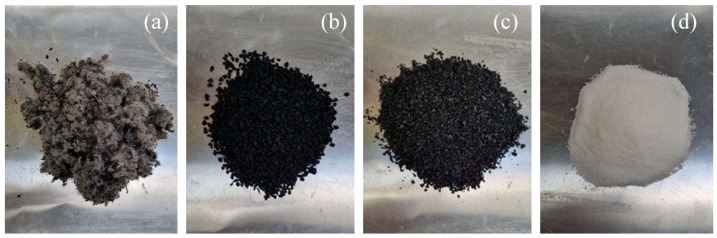
Additives used: (**a**) ELT textile fibre; (**b**) 4 mm ELT rubber; (**c**) 0.5–2.5 mm ELT rubber; (**d**) Sodium Polyacrylate.

Two diverse types of additives were studied. First, sodium polyacrylate and three different forms of end-of-life tyres (ELT), as illustrated in Figure 1.

##### Recycled Materials from End-of-Life Tyres

As mentioned before, the use of end-of-life tyres are a fantastic opportunity to reduce the resources consumption and there are plenty of chances to be included in building materials. In the literature, most of the studies use crumb rubber from end-of-life tyres within 0.075 and 0.6 mm [24,26]. In this paper, three different end-of-life tyre wastes have been used. Two of them are crumb rubber with a size of 0.5–2.5 mm and 4 mm. Recycled textile fibres from ELT were also used as can be seen in Figure 1. The aim is to study how this waste performs better and check its versatility.

##### Sodium Polyacrylate

Table 1 shows super absorbent polymer properties used in this study provided by Alquera, S.L. [32]. This material can retain, and free, vast amounts of water and it has been used as a lightening additive. When mixed with water, its volume increases up to 200–300 times [33], thus producing big voids inside the plaster. Once the plaster is set and dried, particles go back to their original size, leaving air bubbles inside the sample that makes the sample more lightweight.

#### 2.1.3. Dosages and Samples

To achieve the optimal plaster/water ratio, saturation mixing method included in UNE-EN-13279-2 was used [34]. The notation to refer the different dosages use the following code: E-0.7-NAPA-TT, where E refers to Iberyola E-35 plaster, 0.7 is the plaster/water ratio, NAPA is sodium polyacrylate and TT indicate the type of end-of-life tyre used. This can be FG for fine granules, GG for gross granules used and TF for textile fibres. Table 2 shows the different dosages used and the amount of each material. The amount of sodium polyacrylate is determined experimentally until the optimal amount is obtained that does not affect the sudden decrease in the workability of the samples. On the other hand, the recycled materials, due to their low density, were dosed considering their equivalent in volume based on the density of the compound in question. In this way, the same volume ratio of mixture of plaster compounds was always obtained.

All the samples were handmade. First, sodium polyacrylate was mixed with plaster. Then, the end-of-life tyre was mixed either with plaster or water. After that, plaster was mixed according to UNE-EN-13279-2 [34]. All the additives, except textile fibre, were mixed with the plaster before start to mixing. Textile fibre was mixed with the water.

Table 3 shows the different samples prepared for the realisation of these tests. Three samples of each dosage were elaborated. Samples were elaborated following UNE-EN-ISO-13249-2 [34] standard recommendations and then, they were placed at laboratory conditions (23 °C at 50% RH) during a week. Both, Ignition test and acoustic performance samples (25 × 9 × 2 cm^3^) were obtained from 25 × 25 × 2 cm^3^ samples after that tests were conducted.

### 2.2. Physical Characterisation

This characterisation study is divided into two distinct parts. First, a mechanical characterisation is performed. Then, thermal, acoustic and fire performance tests are also conducted. On the one hand, mechanical characterisation was performed in Madrid, at “Escuela Técnica superior de Edificación”. On the other hand, tests for thermal conductivity, sound absorption and fire ignitability were conducted in structures laboratory of Civil Engineering Department, in the University of Coimbra (Portugal).

#### 2.2.1. Mechanical Characterisation

**Figure 2:**
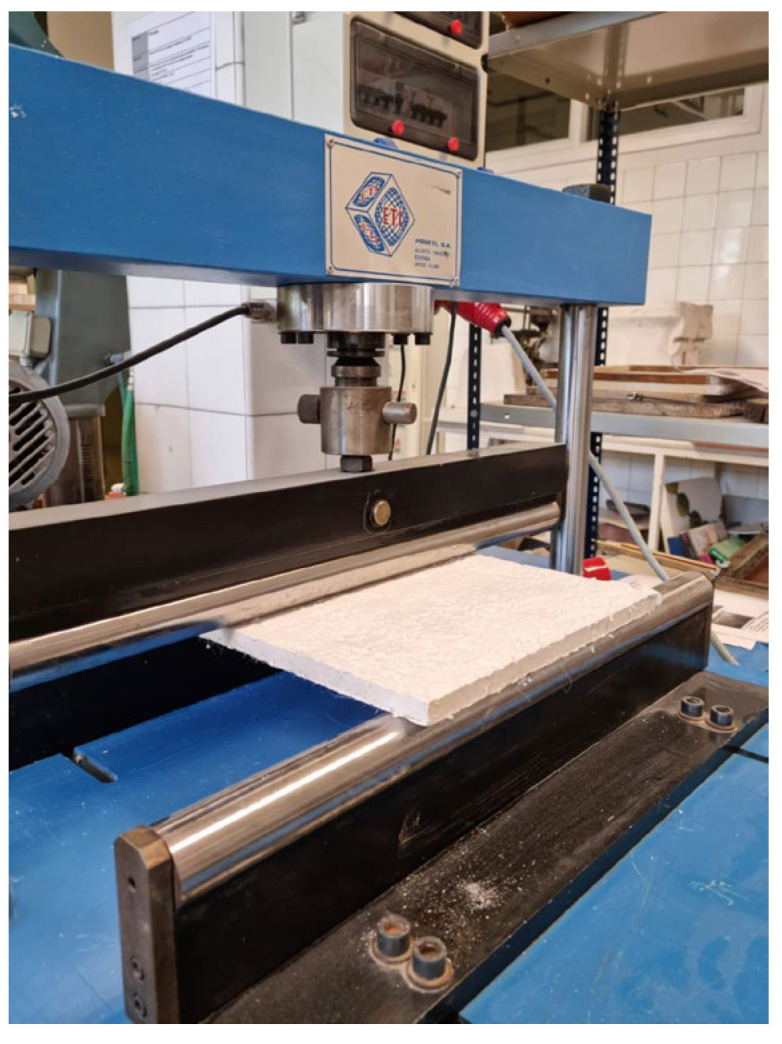
Bending test machine by Proeti, S.A. in Madrid.

**Figure 3:**
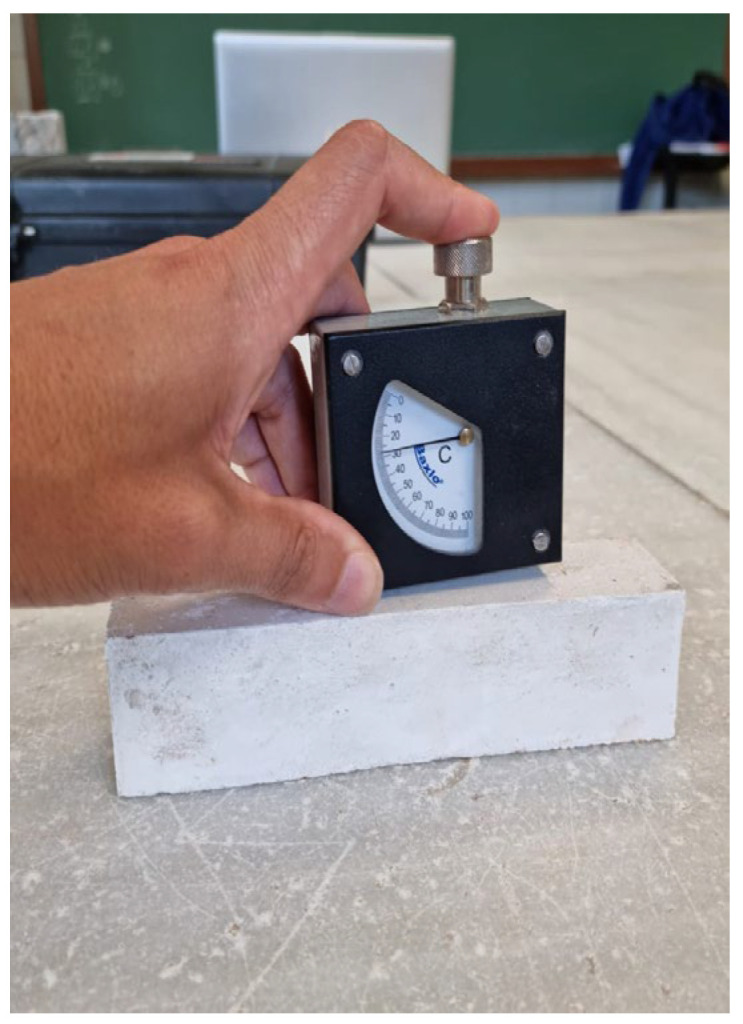
Shore C durometer test.

**Figure 4:**
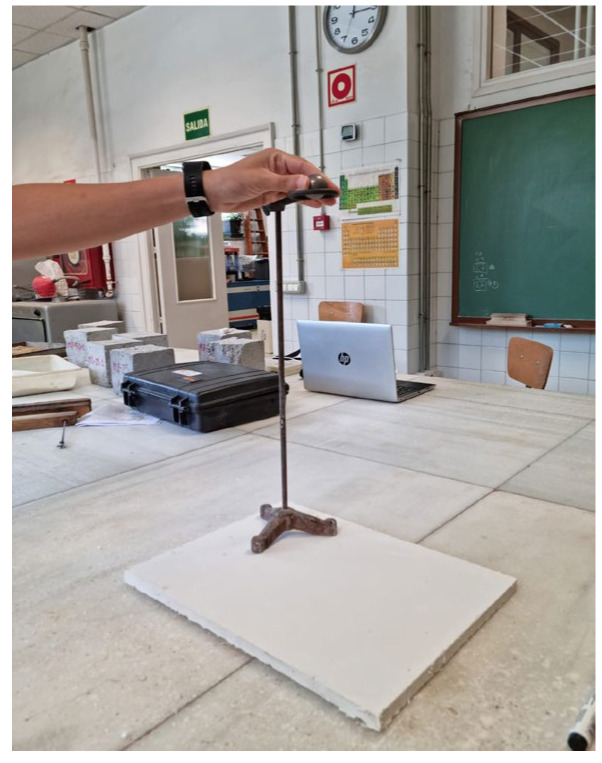
Surface hardness impact test.

Mechanical characterisation was performed with three tests, surface hardness, Impact, and flexural strength tests. Three samples of 500 × 300 × 20 mm^3^ were elaborated following UNE-EN-13279-2 [34]. Flexural strength test was conducted using a Bending test machine from Proeti, S.A. (Madrid) as can be seen in Figure 2. Results were collected and averaged. After that, a surface hardness test was performed according to UNE-EN-12859: 2012 [38]. In this test, plates must stand for 30 min without visible damage signs a bear load of 6 kg on its central fibre (0.1 kN/m) according to UNE-EN 14246:2007 [39]. Regarding Hardness, five measurements in each sample side are taken and averaged using Shore C durometer seen in Figure 3. A complementary surface hardness test from UNE-EN-520: 2005 + A1: 2010 [40] was also performed using bending test samples. Tests were performed measuring the surface footprint caused by the impact of a small steel ball with a diameter of 50 mm and a weight of 510 ± 10 g dropped from a height of 50 cm, as can be seen in Figure 4. Three measurements in each sample are required. Results are averaged and expressed in millimetres. Finally, a non-standardised deformation test was conducted. In this test, 500 × 300 × 20 mm^3^ samples were suspended by the short edges and kept in this position for 4 weeks. After this time, the deformation produced by the self-weight of the plates was measured.

#### 2.2.2. Thermal Conductivity

The thermal conductivity was measured in two separate ways. One using heat flux meter method, and another one using guarded hot plate method. The aim is to set a relationship between the results of these two methods and try to obtain a measurement difference as small as possible. In this way, it is possible to validate both measurement methods.

##### Heat Flux Meter Method

**Figure 5:**
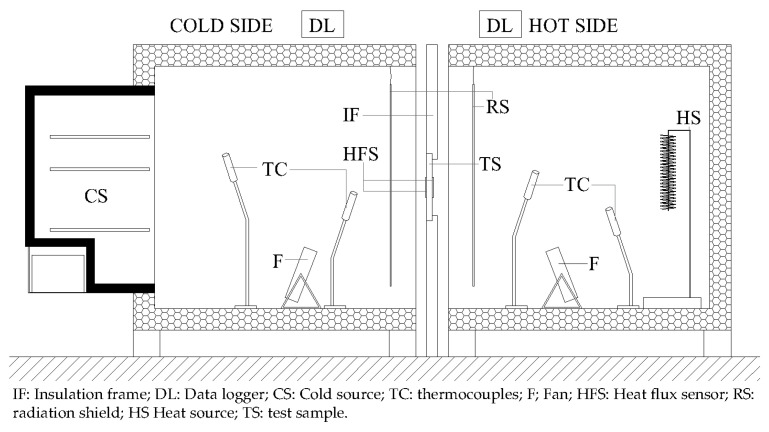
Schematic vertical cross-section of the climatic chambers used for the tests.

The first test was conducted using the heat flux meter (HFM) method. For this, the equipment, as can be seen in Figure 5, is made of two climatic chambers (103 × 106 × 100 cm^3^) insulated with two layers of both, expanded and extruded polystyrene with a total thickness of 100 mm.

**Figure 6:**
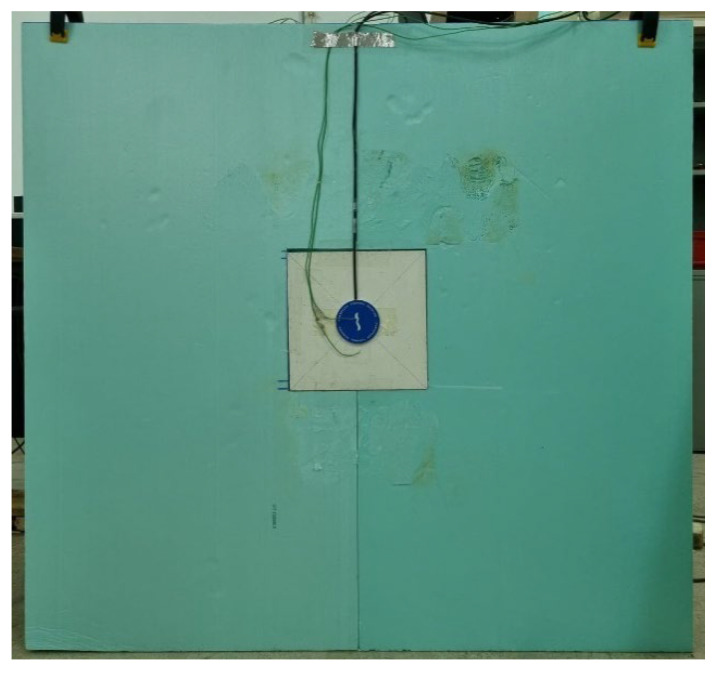
Sample placed in XPS frame.

One chamber is a hot box heated by an electric resistance, and the temperature is set in 40 °C. The other is a cold box cooled by a refrigerator and the temperature is set in 5 °C. As the samples (25 × 25 × 2 cm^3^) are smaller than thermal box, they are fixed in a XPS frame panel, as illustrated in Figure 6. To fix and avoid any air heat transmission through the XPS joint, it has been glued with a non-corrosive glue and then sealed with glue tape.

**Figure 7:**
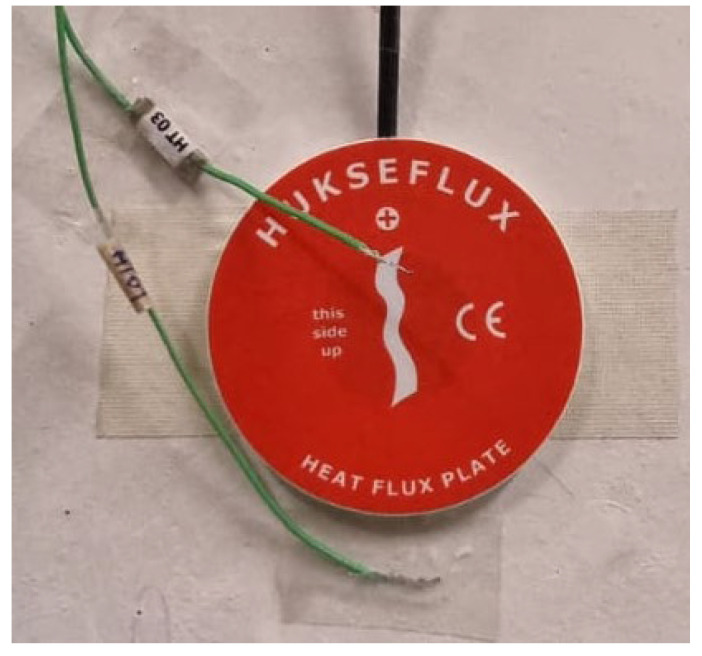
Thermocouple and heat flux meter setup.

After that, the monitoring system is arranged. It is composed of a heat flux meters (HFM) and thermocouples (TC’s). As the size of the sample is reduced, two HFM (Hukseflux model HFP01, precision: ±3%) were used to measure the heat flux in this study. One was set in the hot side, and another in the cold side. Eight type K (1/0.315) PFA insulated thermocouples certified with class 1 precision were used to measure the temperatures. The thermocouples were calibrated within 5–45 °C temperature range with a 5 °C increment. For this, they were immersed in a thermostatic stirring water bath (Heto CB 208). The configuration of the thermocouples, as can be seen in Figure 7, was the following:

Four thermocouples were used in each side of the test sample. One of them (TC1) was placed in the centre of the sample, below the HFM sensor, and measured the sample surface temperature. To measure the air temperature between the surface and the radiation shield (Figure 5), the second was placed in the air, a few centimetres from the surface. Finally, the two remaining thermocouples are placed inside each climate chambers and measured the corresponding hot and chilly air temperature.

Lastly, the equipment precision, measurement range, model, and brand used to take the measurements is shown in Table 4 [41]. Both, heat flux and temperature measurements were recorded by a PICO TC-08 data logger with a precision of ±0.5 °C on each side of the samples. Recorded data were managed by connecting the two data loggers to a computer and using the software provided by PICO, PicoLog v6.1.10. The minimum duration of each experimental test was 8 h, taking measurements continuously. These measurements were taken from the beginning of the test.

##### Guarded Hot Plate Method

**Figure 8:**
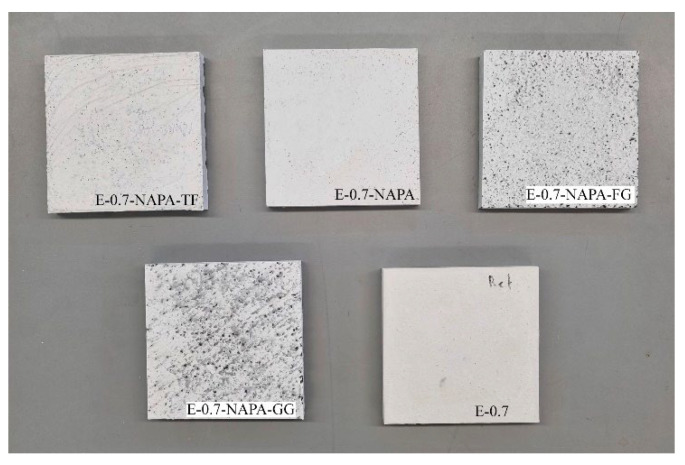
150 × 150 × 20 mm^3^ guarded hot plate samples used.

The second test was conducted using a state-of-the-art thermal conductivity test device, λ-meter EP500e from Lambda-Meßtechnik GmbH, Dresden (Germany). following guarded hot plate method, in accordance with UNE-EN-12664 [35]. Figure 8 shows 15 × 15 × 2 cm^3^ samples that were used to evaluate the thermal conductivity. Two samples of each dosage were tested. Previously, samples were conditioned (23 °C at 50% RH) for over 24 h until they got a nearly constant mass. For this test, it is necessary for the sample’s sides exposed to the flux to be as much flat and parallel as possible. Due to the manual process of samples elaboration, most of them had some superficial irregularities that had to be sanded to assure this parallelism. Thus, the thickness of the samples is slightly lower than 20 mm. Before and after the test, each sample were weighted.

**Figure 9:**
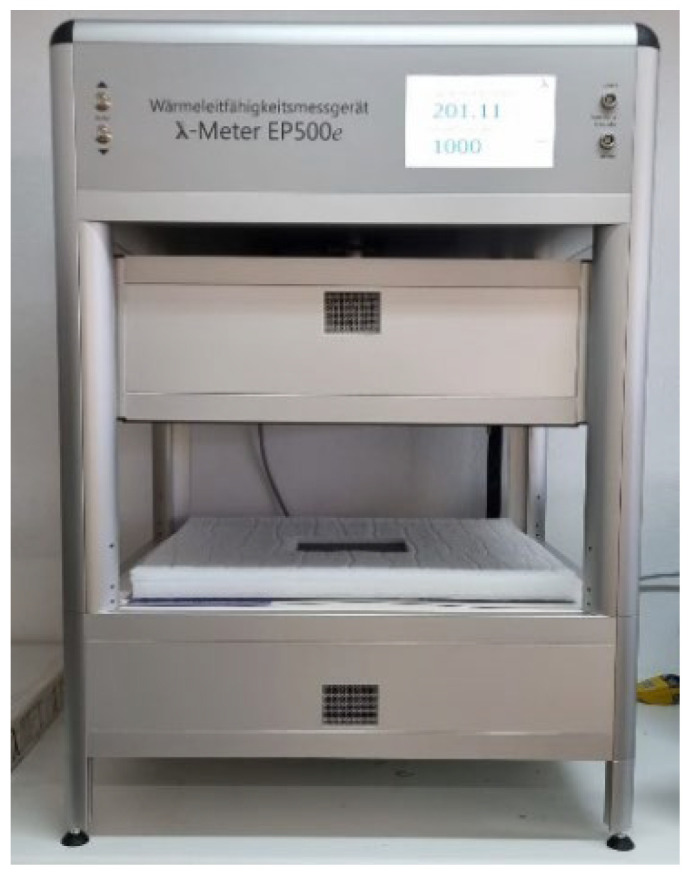
λ-meter machine setup.

After that, the sample was placed in the middle of the lower cold plate. To ensure a unidirectional heat flux and make possible an accurate measurement, an insulation foam was placed around the sample (guard thermal insulation), as can be seen in Figure 9. Once the sample is placed, ***λ***-meter EP500e determine sample’s thickness (***d***) in mm, temperature difference (“**Δ**” ***T***) in Kelvin degrees (or degree Celsius) and heat flow (***Q***) in Watts. With these data and knowing the test sample area (***A***) in m^2^, the thermal conductivity is automatically determined, using the following expression:

(1) $$\lambda =\frac{\dot{Q}\cdot d}{\Delta T\cdot A}\;$$

For this test, the temperature difference between plates (“**Δ**” ***T***) was set to 15 °C, and test pressure to 1000 Pa, according to other studies [40,41]. Due to the manual elaboration of the samples, upper and lower face were not 100% flat. To assure a full contact between the face and the plate and heat flux transmission, two rubber pads were placed between the plates and the samples. Finally, to avoid a possible mass variation due to condensation humidity, samples were covered with a thin polyethylene film.

Measurements were conducted for three average sample temperatures: 10, 25 and 40 °C. Each sample temperature test must have a minimum period of 120 min in which thermal conductivity coefficient should not change more than 1%. After the test, the thermal conductivity was determined, for each average sample temperature. A line was fitted to the three values obtained and the value of the thermal conductivity corresponding to 25 °C (λ25) was taken on the fitted line.

#### 2.2.3. Acoustic Performance

**Figure 10:**
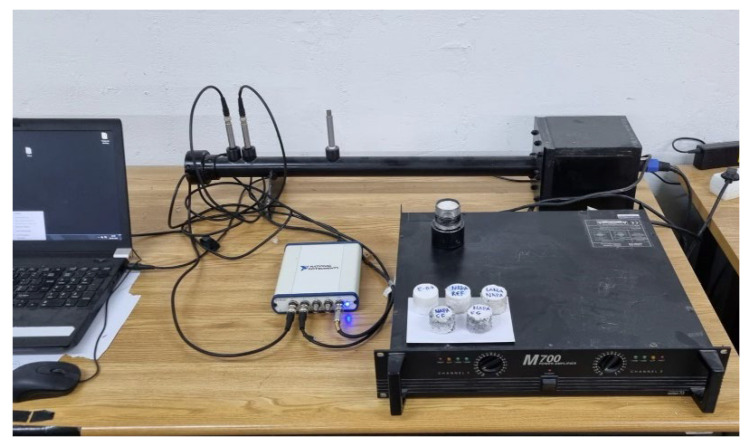
Impedance tube used.

A circular impedance tube (Ø38 mm) showed in Figure 10 was used to measure the sound absorption coefficient. This test was performed in the Acoustic Laboratory of the Civil Engineering Department in the University of Coimbra (PT), according to UNE-EN-ISO-10534-2 [42]. The incidence absorption coefficient was measured by means of the transfer function method, using two microphones. Impedance tube used had a frequency range from 100 Hz to 5000 Hz. Intrinsic acoustic properties were obtained and validated in literature [43,44].

The sound pressure was measured using two G.R.A.S. Sound & Vibration 46 AE 1/2″ CCP free field microphones, a NI USB 4431 acquisition system from Coimbra, Portugal, and the pressure data processed in MATLAB [45].

**Figure 11:**
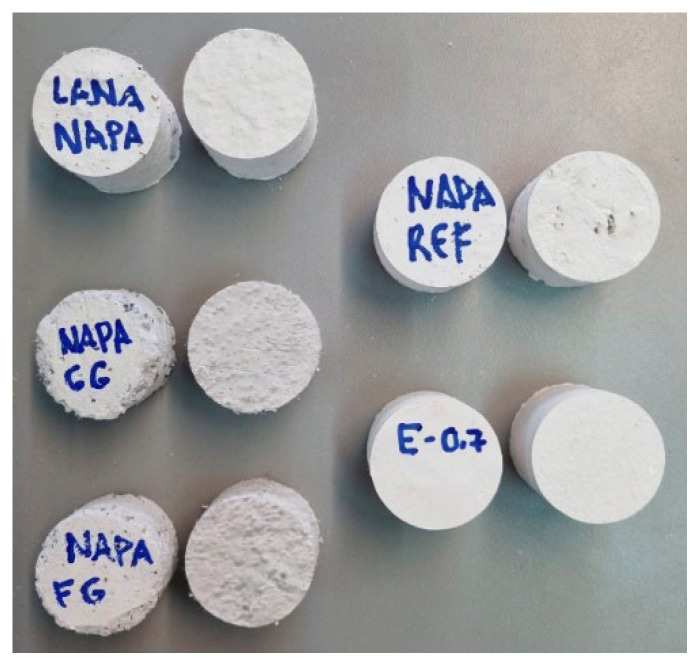
Samples tested.

Samples showed in Figure 11 were prepared. As they were handmade, they needed to be sanded to assure flatness in the sound-exposed face. After this preparation, they were tested. First, environmental parameters of the room were measured (temperature, relative humidity, and atmospheric pressure) and microphones were calibrated. Samples were placed in one side of the impedance tube. As the sample must fill all the surface, Vaseline was used to cover imperfections/voids on the sample’s borders. After, the sample was placed in the sample holder, the sound pressure was measured, and the transfer function calculated.

**Figure 12:**
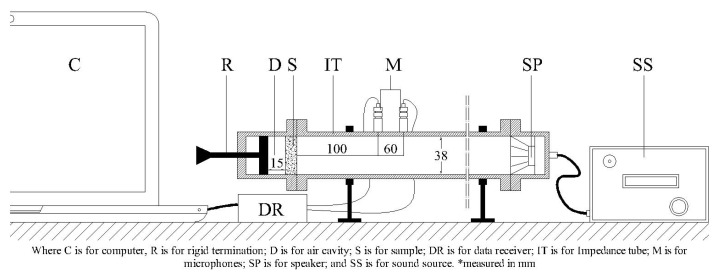
Impedance tube longitudinal cross-section D = 15 mm.

A white noise signal provided by a speaker was generated from the NI USB 4431 to excite the speaker from the OR34 Compact Analyzer. Two microphones B&K Type 4188 1/2″ positioned at 16 cm and 10 cm from the sample surface were used to measure sound pressure microphones. After the measurement, MATLAB was used to process pressure data to obtain sound absorption coefficients. Figure 12 shows experimental setups representation where D = 15 mm is for air cavity thickness.

As mentioned, ISO 10534-2 standard procedure is used, and it is based on three measurements of the same sample. In each measurement, air cavity depth leads to a preservation of the method’s validation. The three measurements are averaged and, with data collected, reflection factor (*r*) is given by Equation (2):

(2) $$r=\frac{P_{r}}{P_{i}}\;$$

where *P_r_* is the reflected pressure (Pa), and *P_i_* is the incident pressure (Pa).

Then, sound absorption coefficient (α) is obtained by Equation (3):

(3) $$\alpha =1-{\left|r\right|}^{2}$$

After that, for its application in buildings, the mean absorption coefficient in building is given by the following Equation (4):

(4) $$\alpha_{m}=\frac{\alpha_{500}+\alpha_{1000}+\alpha_{2000}}{3}\;$$

where $\alpha_{500},\;\alpha_{1000},\;and\;\alpha_{2000}$ are acoustic absorption values for frequencies 500, 1000, and 2000 Hz, respectively.

#### 2.2.4. Fire Ignitability

**Figure 13:**
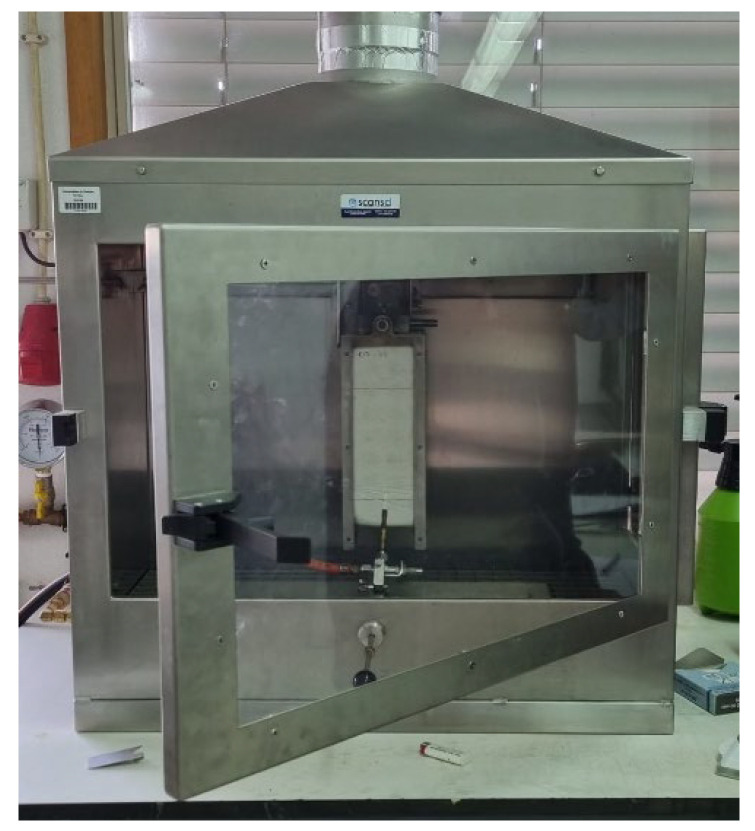
Fire ignitability test machine.

As these studied materials are thought to be used in false ceilings, a fire reaction test with a small ignition tool was also carried out in this study. This equipment, illustrated in Figure 13, is used to perform the Euroclass test, which measure the ignitability of a product when it is subjected to direct impingement of a small flame. It is relevant to the classification of a product into classes B, Bfl, C, Cfl, E, and Efl for all buildings’ materials. This test was performed according to the UNE-EN-ISO-11925-2 [46], in the Fire Lab of the Civil Engineering Department of the University of Coimbra (PT).

**Figure 14:**
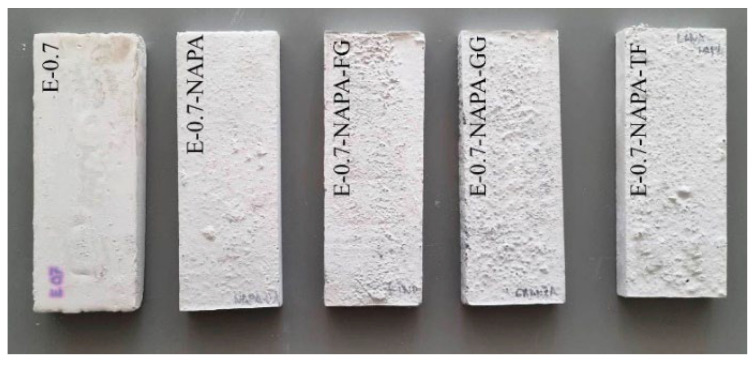
90 × 250 × 20 mm^3^ Fire samples.

**Figure 15:**
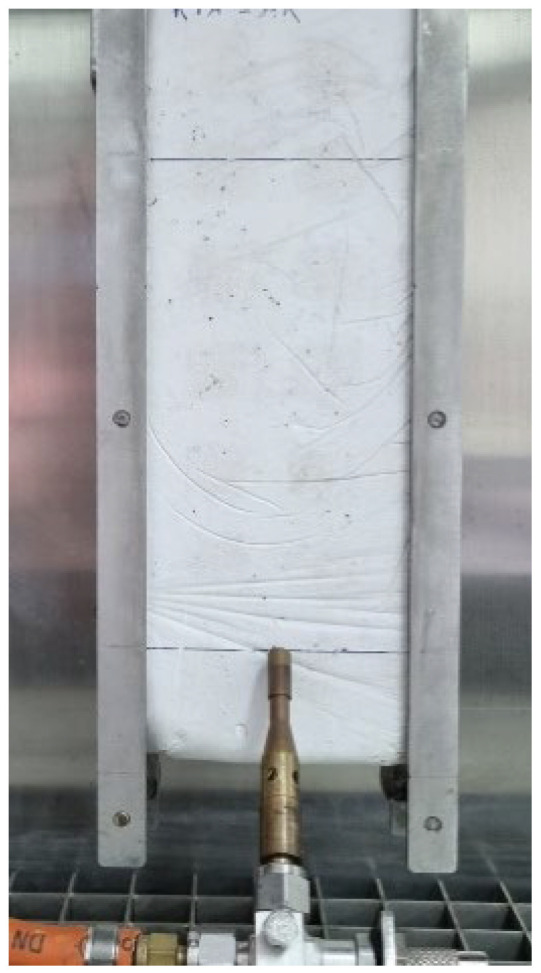
Flame position mark.

Figure 14 shows the 9 × 25 × 2.5 cm^3^ samples elaborated, one of each dosage. Samples were conditioned during a week (23 °C at 50% RH) for over 24 h, until they got a constant mass. The test procedure was the following. First, the samples were weighted and measured. After that, a line was drawn four centimetres from the bottom of the sample, and another fifteen centimetres up the first line. Then, a needle marked the correct flame position. This is in the middle of the bottom line, as can be seen in Figure 15. Once sample position was right, flame was lighted according to the standard. Following this, the flame was applied in a 45° angle during thirty secs. After these thirty secs, the flame was removed, and the sample stayed another thirty secs to ensure that there was no ignition and fire propagation.

**Figure 16:**
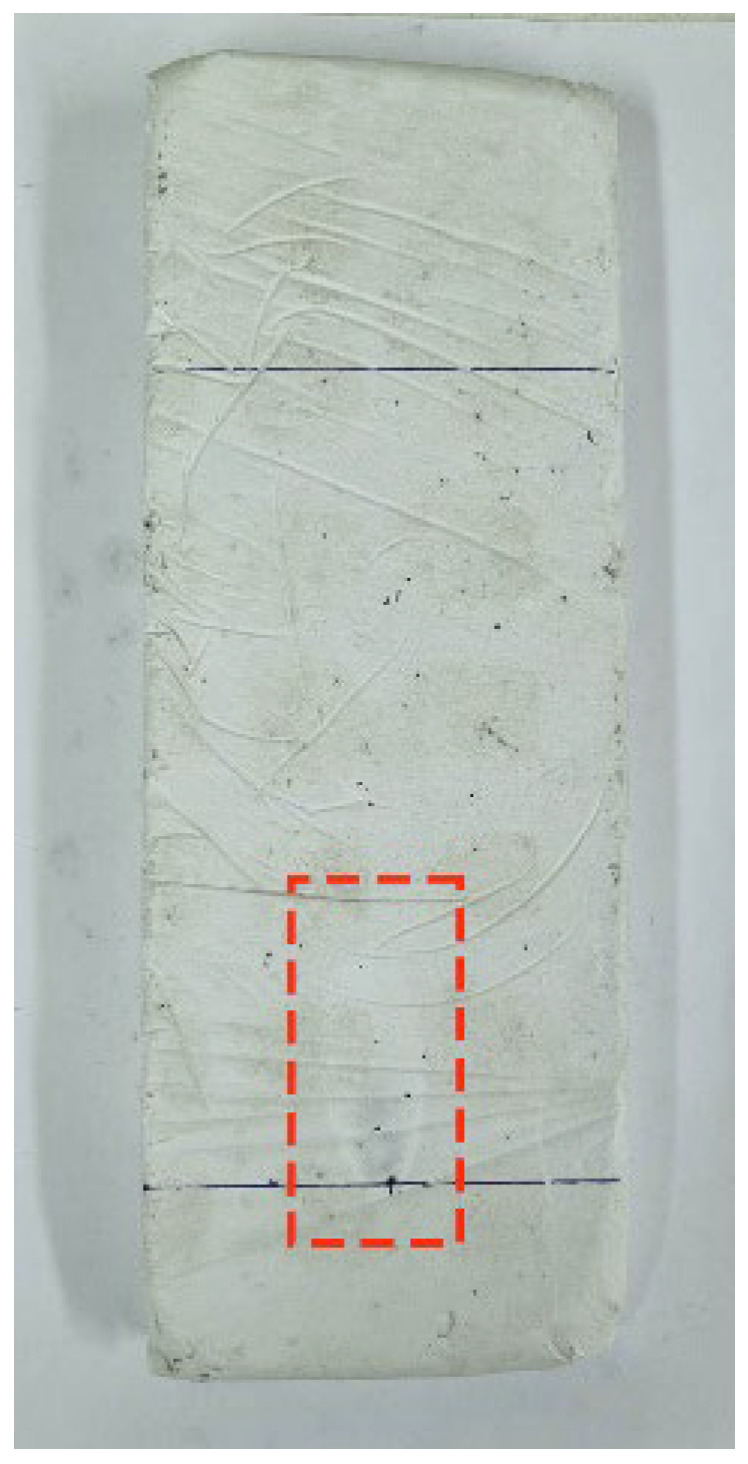
Flame mark length.

After the test, samples were weighed again to see if there was any mass loss. The sample passes the test if the length of flame mark does not reach the other above line, as can be seen in Figure 16.

## 3. Results and Discussion

This section shows the results obtained for the different characterisation tests performed in this research work. To compare all results obtained, E-0.7 has been taken as the reference.

### 3.1. Mechanical Characterisation

**Figure 17:**
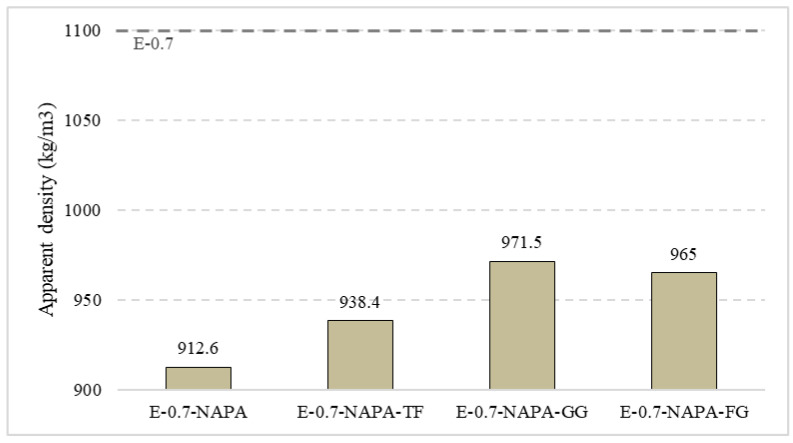
Apparent density (kg/m^3^).

First, mechanical characterisation tests were performed, where density, flexural strength, and hardness were studied. Figure 17 shows density values achieved in this experimental campaign. The polymer and ELT waste led to a decrease in the apparent density of all the samples up to 17%. This density loss is due to the big air holes resulting from the polymer. During the mixing, the polymer increases his volume up to 300 times, taking up more space and reducing gypsum content. Once dried, the polymer returns to its original size making this material more porous. Despite ELT density is smaller than gypsum, ELT wastes additive samples performed worse than E-0.7-NAPA. The best ELT sample was E-0.7-NAPA-TF, which decreased its density up to 17%, being closer to E-0.7-NAPA.

Types of additives are also related to flexural strength. Table 5 shows how the use of additives led to a flexural strength decrease in all samples but 0.7-NAPA-TF, (0.34 kN) which performed better than E-0.7 (0.32 kN) reference sample. The main difference between both polymer and ELT rubber in their performance is that the polymer does not interact with gypsum during the test, as it returns to original size when samples are dried. ELT affects directly to their mechanical behaviour because there is no cohesion between gypsum and rubber and, despite of his higher tensile strength, their different elasticity leads to a worse performance of this mixture [47]. ELT fibre tensile strength is also higher than gypsum, but there are better inner cohesion and achieve better and more homogeneous mixture. This, lead E-0.7-NAPA-TF to obtain the best results of all samples performed All composites got flexural strength values over UNE-EN 12,859 [39] standard requirements (0.18 kN).

Surface hardness test and deformation results are analysed in Table 6. E-0.7 (86 Shore C units) results were worse than E-0.7-NAPA-TF (88 Shore C units), and E-0.7-NAPA (89 Shore C units). This is due to the more water/plaster ratio, the less shore C hardness achieved [48]. As the polymer can absorb vast amounts of water from the mixture, E-0.7-NAPA (89 Shore C units) achieved the best performance. When ELT textile fibre is added, this result decreases as this fibre also retains water. Samples with rubber, E-0.7-NAPA-GG and E-0.7-NAPA-FG achieved the worst results as rubber mixed with gypsum, weakening the final composite.

However, results are practically inversed in the surface hardness impact test. Samples without rubber achieved a worse result than those with rubber. This is due to rubber elasticity [49], which allows it to absorb and reduce the load transmission trough the sample. With this, rubber size is also determinant, as can be seen. With a smaller size of rubber, load absorption capacity increases, as the load is more homogeneously distributed. In this test, E-0.7-NAPA-FG demonstrated the smallest footprint with 7.1 mm, compared to E-0.7, with 9 mm and achieved the worst result. Finally, deformation results demonstrate almost irrelevant deformation of the samples. As can be seen, all samples achieved smaller values than E-0.7 (12 μm), which was the highest deformation. E-0.7-NAPA-TF achieved the best result (9 μm) because fibre leads the composite to a more homogeneous distribution and collaborate as well as in flexural strength to a good behaviour. On the other hand, samples with rubber, E-0.7-NAPA-GG (42 μm) and E-0.7-NAPA-FG (40 μm) performed worse because of the lack of cohesion inside the sample and rubber elasticity.

### 3.2. Thermal Conductivity

In this section, both hot guarded plate and heat flux meter methods results are first presented and analysed. Then, they are compared.

Table 7 presents the samples conductivity and thermal resistance values obtained from the guarded hot plate tests performed. ELT rubber sample was also evaluated to get a reference value. This will allow analysis of the new composites behaviour.

A small mass variation can be observed in E-0.7 (0.02%), E-0.7-NAPA (0.02%), and E-0.7-NAPA-GG (0.04%). Although samples were covered with a thin plastic film, this variation is due to a water absorption from the cold plate, due to plates temperature. This small mass variation during the test caused by this water absorption was not significant and had no effect on the result.

**Figure 18:**
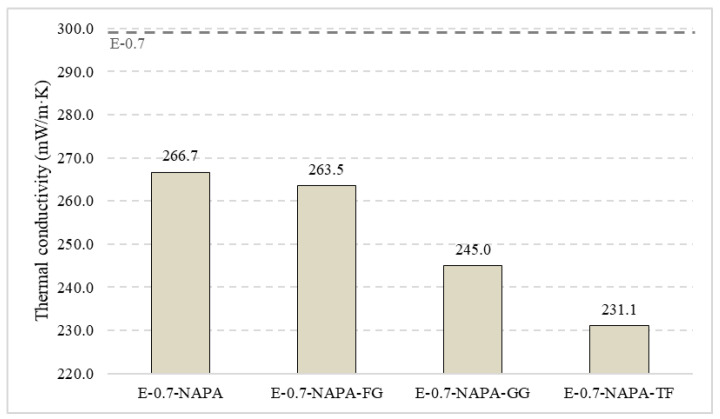
Guarded hot plate test thermal conductivity results (mW/(m·K)).

Figure 18 shows the values achieved by the samples in guarded hot plate test performed. The additive of Sodium Polyacrylate decreased thermal conductivity up to 8%, from 290.1 to 266 mW/(m·K). Therefore, the addition of ELT rubber and textile fibre led to a decrease in all the samples thermal conductivity studied. The best value achieved was 231.1 mW/(m·K) in E-0.7-NAPA-TF, with a 20% reduction. Samples with ELT rubber performed worse than those with textile fibre. This is due to the higher mass presence and density into the matrix. The worst ELT additive sample value was obtained by E-0.7-NAPA-GG, which got a 9% decrease with respect to the reference. That means that the smaller rubber size, the higher conductivity value achieved.

**Figure 19:**
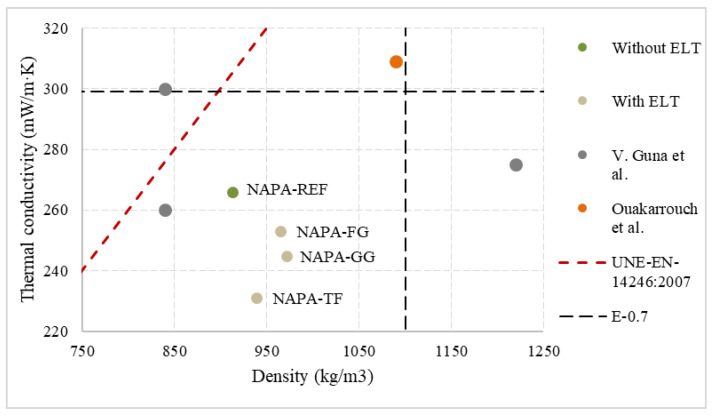
Dispersion comparative thermal conductivity results.

To emphasise the results obtained, Figure 19 shows a comparison of different values obtained by different authors in the literature as well as UNE-EN-14246: 2007 [39] standard specifications. Studies compared have been selected for two reasons. First, the use of both natural and synthetic lightweight wastes. Then, to avoid mistakes in measurement comparison, studies compared used hot guarded plate method to determine thermal conductivity.

Values obtained by those samples with ELT additives are close to each other. As a main advantage, density is lower than reference in all samples, and all of them are behind standard limits. The study performed by Guna et al. used natural wool and coir fibre to develop thermal and moisture resistant ceiling tiles gypsum based [11]. This study recorded 840 kg/m^3^ samples densities, which is a low density. However, results are like the reference, and all of them are above those with ELT additive. The best value achieved is 260 mW/(m·K), and only E-0.7-NAPA performed worse. Ouakarrouch et al. developed a new light plaster by adding animal feathers. In this case, values obtained were worse than all compared values [50].

**Figure 20:**
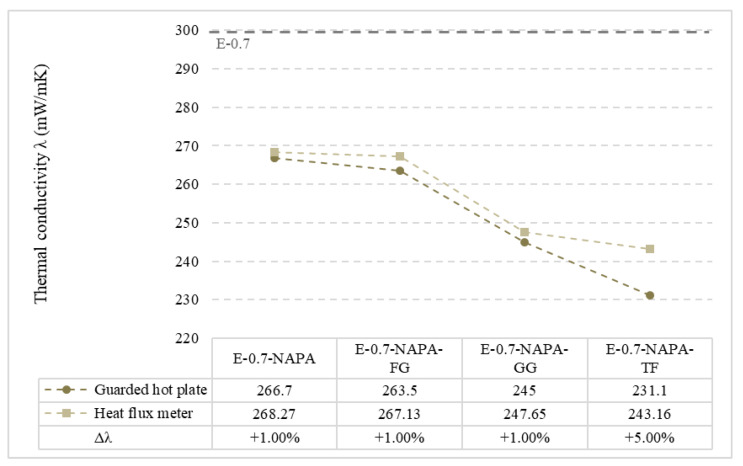
Measured thermal conductivity values comparison (mW/(m·K)).

On the other hand, heat flux meter tests results are presented in Table 8. Aligned with Hot guarded plate test results, E-0.7-NAPA-TF was the sample which performed better with a 16% improvement in thermal conductivity value. As commented in guarded hot plate test results, the addition of ELT rubber and textile fibre decreased thermal conductivity values up to 16%. In these tests, values achieved were different than those achieved in lambda meter tests. To validate the two methods employed, a comparison of both heat flux meter and hot guarded plate methods results are presented in Figure 20.

First, all samples but E-0.7-NAPA-TF demonstrated 1% difference thermal conductivities values with respect to guarded hot plate test results. This is due to a homogeneous distribution of different additives present in the samples. Rubber and sodium polyacrylate have a constant mass and volume. This led to a more homogeneous distribution in the mixture. The main difference is noticed in E-0.7-NAPA-TF with a 5% difference. This could be due to two main factors. On the one hand, despite textile fibre is distributed along the sample, due to its shape, it is not completely homogeneous. On the other hand, the Lambda-Meter machine measures the entire 150 × 150 mm^2^ surface, while heat flux meter measures a local region. This fact could directly affect the thermal conductivity measurements as it is not possible to ensure a textile fibre homogeneous distribution. Even so, difference values achieved ensures an enough accuracy to validate heat flux meter method and the test apparatus used as a valid test to characterise thermal conductivity.

### 3.3. Sound Absorption Coefficient

**Figure 21:**
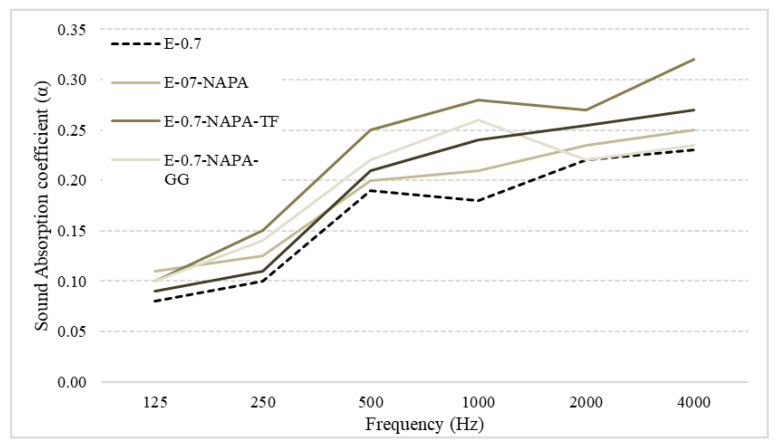
Sound absorption Coefficient (α) test results.

Figure 21 shows the measured sound absorption coefficient. These results show that the addition of both, Sodium polyacrylate and ELT wastes (rubber and textile fibre) improved in a significant way this material characteristics in all octaves studied. The most significant difference occurs at 1000 Hz and 4000 Hz. At 2000 Hz, results remain the same. From 125 to 500 Hz, the results agree with the literature, which marks a deficient performance in these frequencies [51]. The highest sound absorption was reached by E-0.7-NAPA-TF at 4000 Hz (0.32). E-0.7 demonstrated the worst performance in all frequency octaves.

Finally, a mean value of each sample is obtained and shown in Table 9. It should be noticed that all samples studied improved acoustic performance. E-07-NAPA improved 9.3% and main improvement was in 2000 Hz frequency. After that, the addition of ELT rubber led to an increase up to 19.5% (from 0.20 to 0.24), depending on the rubber size. Lastly, ELT textile fibre achieved a 35.6% improvement, which makes E-07-NAPA-TF the sample who performed better. The best improvements are achieved in 1000 Hz, where sound absorption coefficient difference is up to 0.1. This frequency range (500–2000 Hz) includes most of the main sounds, such as the human voice.

### 3.4. Fire Performance

According to fire standard UNE-EN-11925:1 [46], contained in UNE-EN-14246 standard [39], Table 10 shows the fire ignitability test results performed. Neither gypsum nor sodium polyacrylate are flammable materials, unlike ELT wastes [26,47]. Initial weight was set to check any mass loss during the test.

This section may be divided by subheadings. It should provide a concise and precise description of the experimental results, their interpretation, as well as the experimental conclusions that can be drawn.

**Figure 22:**
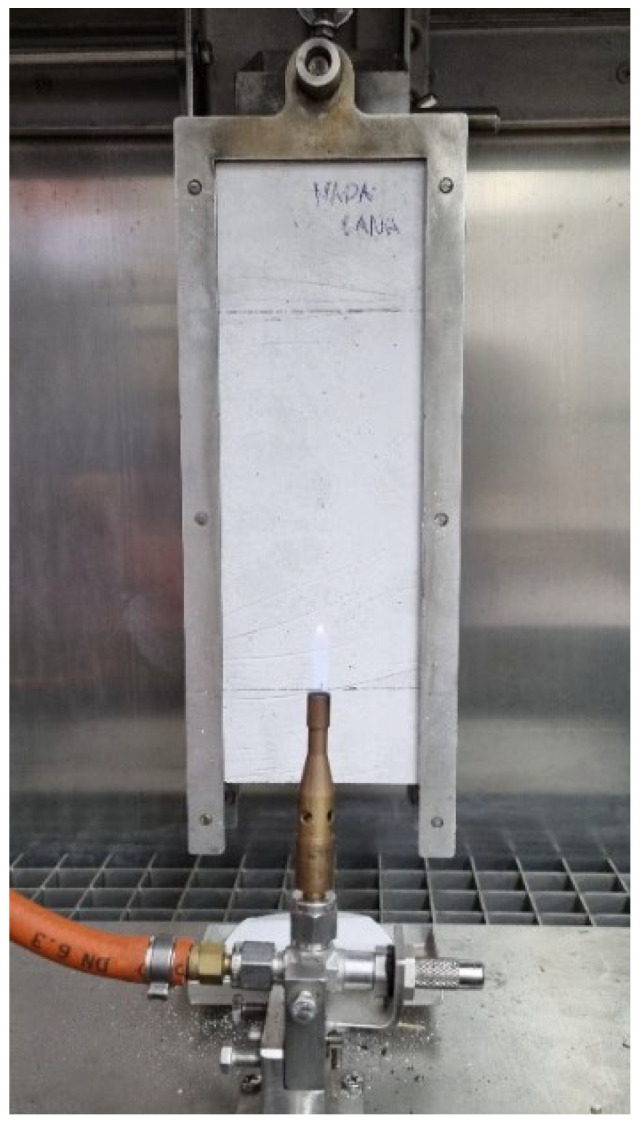
Ignitability test machine set up (UNE-EN-11925:1).

A negligible mass variation was registered in plaster samples due to superficial gypsum dust that fell after removed from the sample holder shown in Figure 22. In all cases, this mass variation was around 0.1–0.2 g, a completely negligible value that does not affect to the test. ELT rubber sample mass did not change. Despite ELT’s flammability, none of the samples ignited. This is due to two main reasons.

**Figure 23:**
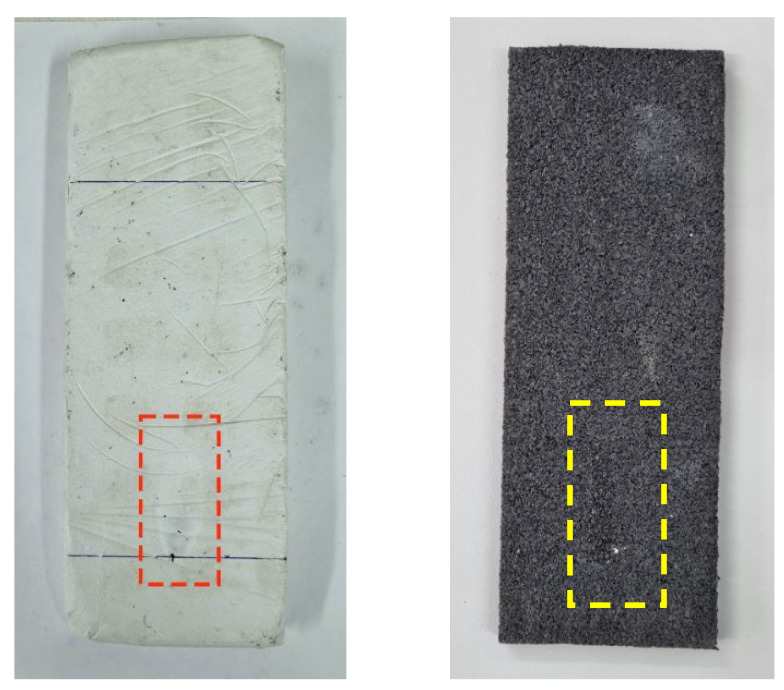
E-0.7-Na-TF and ELT rubber samples after fire test.

This test applies a flame for thirty secs. To get ignited, a longer flame application time is required. In addition, in samples with ELT rubber and textile, this material is encapsulated within the gypsum, making combustion even more difficult. Regarding flame length, it was almost the same in all the samples, but in ELT rubber sample, which reached 55 mm. The mark set by the fire in the samples was small and almost invisible, as can be seen in Figure 23. To totally classify these materials as Class B, UNE-EN-13823 [52] complementary test would be necessary to perform as exposed in UNE-EN-13501-1 [53].

### 3.5. Environmental Impacts: Global Warming Potential

Finally, a global warming potential analysis, was performed to determine these composites elaboration environmental impact. As far as end-of-life tyre is used in this study as the main additive, it is a great opportunity to reduce environmental impact produced by this great waste. To perform this analysis, it is necessary to determine what is functional unit (FU) in each material. For this environmental product declaration of E-35, gypsum [54] and tyres [55] were used. These documents show the environmental cost of these products. In this studio, ranges considered were from A1 to A3 for E-35, and C1–C4 for tyres. Sodium polyacrylate (NAPA) information referred to this topic is not available after consulting with supplier business. The aim of this comparison is to set a potential reduction of main resources and how it potentially reduces environmental impact.

Table 11 shows analysis conducted, and values of potential global warming potential reduction achieved. It can be noticed that only with sodium polyacrylate additive, a 22% decrease of CO_2_ emissions is achieved. This is due to a smaller E-35 quantity used. End-of-life tyre additive also got a reduction of this global warming potential. Highest CO_2_ decrease was achieved in E-0.7-NAPA-FG and E-0.7-NAPA-GG, with a 34% CO_2_ potential reduction. Both achieved the same reduction as they have the same quantity of material in the mixture. The use of textile fibres reached only a 20% CO_2_ potential reduction, but it is only due to the smaller quantity of this material with respect to E-0.7-NAPA-FG and E-0.7-NAPA-GG. With these results it is possible to confirm that the use of ELT recycled materials reduces in a significant way the final product environmental impact (GWP).

## 4. Conclusions

End-of-life tyre waste is one of the most contaminating materials existing nowadays. In view of this, its use as additive in building materials represents a potential a solution to this problem and a new life improving some of the required characteristics in this sector. In this study, recycled granulated rubber, and recycled textile fibres from end-of-life tyres are presented as additive to develop false ceiling gypsum plates. Thus, this study achieved important results related to this additive, as highlighted next:

- The addition of both ELT recycled rubber and textile fibre decreased material density up to 15%. This leads to a better material elaboration workability, easier transport, and assembly;
- The addition of sodium polyacrylate improves thermal conductivity up to 8% with respect to the reference. Therefore, the addition of ELT rubber and textile fibre decreases thermal conductivity. Thus, textile fibre was the additive which performed better in this characteristic;
- Thermal conductivity values obtained from both guarded hot plate and heat flux meter methods were strongly related, with differences around 1%, having only one exception (5% for the ELT textile fibres), which is also a remarkable result;
- Acoustic absorption values showed a significant improvement in all samples studied with respect to the reference. However, it could perform even better if samples were perforated. This would change samples aesthetic and that is the main reason it was not done in this study;
- Ignitability test resulted in no ignition, nor a significant mass reduction. Due to this, and according to UNE-EN-13501-1 [53], samples can be classified as “Class E”;
- Finally, regarding environmental behaviour, only by reducing the amount of matrix material (gypsum/plaster) can a reduction of energy and resources consumption be achieved. In this study, the use of ELT recycled rubber and textile fibres can reduce up to a 34% of CO_2_ emissions with respect to the reference.

Plaster-based composites are highly studied, but there are few studies in the literature with the addition of ELT wastes, rubber, and textile fibre. In this study, results shows that the elaboration of new plaster-based material with these wastes still have a lot of chances and ways to be studied.

## Figures and Tables

**Table 1:** Sodium Polyacrylate physical properties [32].

Physical Property	Units	Values
**Appearance**	-	Granulated, white
**Particle Size**	µm	150–850
**Apparent Density**	g/mL	0.5–0.75
**Humidity**	%	≤5
**pH (1% gel in 0.9% NaCl)**	-	6.0 ± 0.5
**Ignitability**	-	No flammable

**Table 2:** Dosages composition of the different plaster materials.

Notation	E-35	Water	Sodium Polyacrylate	ELT Recycled Materials
**E-0.7**	1000	700	-	-
**E-0.7-NAPA**	1000	700	15	-
**E-0.7-NAPA-TF**	980	686	15	20
**E-0.7-NAPA-GG**	600	420	9	168.7
**E-0.7-NAPA-FG**	600	420	9	168.7
Weight in grams				

**Table 3:** Samples elaborated and tests performed.

Samples	Tests
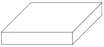 15 × 15 × 2 cm^3^	To determine thermal conductivity by guarded hot plate method. For this, λ-Meter EP500e from Lambda-Meßtechnik GmbH Dresden (Germany) was used, in accordance with the standards UNE-EN-12664 [35] and UNE-EN-1946-2 [36]
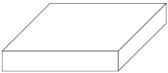 25 × 25 × 2 cm^3^	Determination of the coefficient of material thermal conductivity using the heat flux meter (HFM) method. This test follows UNE-EN 12,664 [35] and UNE-EN-1946-3 [37] standard proceedings.
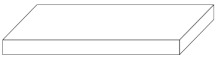 50 × 30 × 2 cm^3^	Surface Hardness (Shore C), and Pure bending test to determine the suitability of the material for use in precast. For this, Proeti, S.A., (Madrid, Spain) ending test equipment was used and the recommendations of the standard UNE-EN 12859: 2012 [38] were followed.

**Table 4:** Equipment used in the HFM thermal conductivity tests [41].

Equipment	Brand	Model	Measurement Range	Precision
**Thermocouple**	LabFacility	Type K * (1/0.315)	−75 to + 260 °C	±1.5 °C
**Heat flux meter**	Hukseflux	HFP01	−2 to + 2 kW/m^2^	±3%
**Data-logger**	PICO	TC-08	−270 to + 1820 °C	±0.5 °C

* Tolerance class 1 certified.

**Table 5:** Bending test results.

Sample		Maximum Breaking Load (kN)	Mean Value Load (kN)	Pass UNE-EN-14246 Requirements (6 kg, 30′)
**E-0.7**	1	0.35	0.32	YES
	2	0.3		YES
	3	0.32		YES
**E-0.7-NAPA**	1	0.3	0.30	YES
	2	0.29		YES
	3	0.3		YES
**E-0.7-NAPA-TF**	1	0.34	0.34	YES
	2	0.32		YES
	3	0.35		YES
**E-0.7-NAPA-GG**	1	0.27	0.27	YES
	2	0.26		YES
	3	0.28		YES
**E-0.7-NAPA-FG**	1	0.29	0.29	YES
	2	0.28		YES
	3	0.29		YES

**Table 6:** Surface hardness tests results.

Sample	Hardness (Shore C)	Surface Hardness Impact (mm)	Deformation (μm)
**E-0.7**	86	9.0	12
**E-0.7-NAPA**	89	8.1	11
**E-0.7-NAPA-TF**	88	7.7	9
**E-0.7-NAPA-GG**	79	7.2	42
**E-0.7-NAPA-FG**	81	7.1	40

**Table 7:** Guarded hot plate tests results.

Sample	Weight Before [g]	Weight After [g]	Δ Weight (%)	Conductivity [mW/(m·K)]
**ELT RUBBER**	178.7	178.7	0.00%	122.4
**E-0.7**	486.7	486.8	0.02%	290.5
**E-0.7-NAPA**	415.8	415.9	0.02%	266.1
**E-0.7-NAPA-TF**	448.1	448.1	0.00%	231.1
**E-0.7-NAPA-GG**	475.5	475.7	0.04%	245.0
**E-0.7-NAPA-FG**	458.2	458.2	0.00%	263.5

**Table 8:** Heat flux meter measured values.

Sample	Heat Flux (Average) (W/m^2^)	Thickness (mm)	ΔT(K)	Conductivity [W/(m·K)]
**E-0.7**	128.6	19.5	8.43	297.65
**E-0.7-NAPA**	115.7	19.5	8.41	268.27
**E-0.7-NAPA-TF**	108.4	19.0	8.47	243.16
**E-0.7-NAPA-GG**	114.7	19.0	8.80	247.65
**E-0.7-NAPA-FG**	114.4	19.2	8.22	267.13

**Table 9:** Mean sound absorption coefficients.

Samples	Frequencies (Hz)			Mean Value	Δ(α)
	500	1000	2000		
**E-0.7**	0.19	0.18	0.22	0.20	-
**E-0.7-NAPA**	0.20	0.21	0.24	0.22	+9.3%
**E-0.7-NAPA-TF**	0.25	0.28	0.27	0.27	+35.6%
**E-0.7-NAPA-GG**	0.22	0.26	0.22	0.23	+18.6%
**E-0.7-NAPA-FG**	0.21	0.24	0.26	0.24	+19.5%

**Table 10:** Fire Ignitability test results.

Sample	Weight		Ignited	Particle Presence	Flame Length (mm)
	Before Test (g)	After Test (g)			
**ELT rubber**	186.7	186.7	NO	NO	55
**E-0.7**	699.4	699.3	NO	NO	35
**E-0.7-NAPA**	663.1	662.8	NO	NO	32
**E-0.7-NAPA-TF**	606.4	606.3	NO	NO	36
**E-0.7-NAPA-GG**	615.8	615.6	NO	NO	35
**E-0.7-NAPA-FG**	650.8	650.8	NO	NO	34

**Table 11:** Global warming potential analysis.

Samples	E-35 (kg)	Water (kg)	S. Pol. (kg)	TF (kg)	FG (kg)	GG (kg)	CO_2_ (kg/m^3^)	Δ CO_2_ (%)
**E-0.7**	647.06	452.94	-	-	-	-	45.29	-
**E-0.7-NAPA**	530.46	371.32	10.83	-	-	-	37.13	−22%
**E-0.7-NAPA-TF**	539.06	377.34	11.00	11.00	-	-	37.73	−20%
**E-0.7-NAPA-GG**	481.60	337.12	10.84	-	-	135.45	33.94	−34%
**E-0.7-NAPA-FG**	481.60	337.12	10.84	-	135.45	-	33.71	−34%

## Data Availability

Not applicable.
